# Eic1 links Mis18 with the CCAN/Mis6/Ctf19 complex to promote CENP-A assembly

**DOI:** 10.1098/rsob.140043

**Published:** 2014-04-30

**Authors:** Lakxmi Subramanian, Nicholas R. T. Toda, Juri Rappsilber, Robin C. Allshire

**Affiliations:** 1 Wellcome Trust Centre for Cell Biology, Institute of Cell Biology, School of Biological Sciences, The University of Edinburgh, Edinburgh EH9 3JR, UK; 2 Institute of Bioanalytics, Department of Biotechnology, Technische Universität Berlin, 13353 Berlin, Germany

**Keywords:** CENP-A, centromeres, epigenetics, fission yeast, Mis18

## Abstract

CENP-A chromatin forms the foundation for kinetochore assembly. Replication-independent incorporation of CENP-A at centromeres depends on its chaperone HJURP^Scm3^, and Mis18 in vertebrates and fission yeast. The recruitment of Mis18 and HJURP^Scm3^ to centromeres is cell cycle regulated. Vertebrate Mis18 associates with Mis18BP1^KNL2^, which is critical for the recruitment of Mis18 and HJURP^Scm3^. We identify two novel fission yeast Mis18-interacting proteins (Eic1 and Eic2), components of the Mis18 complex. Eic1 is essential to maintain Cnp1^CENP-A^ at centromeres and is crucial for kinetochore integrity; Eic2 is dispensable. Eic1 also associates with Fta7^CENP-Q/Okp1^, Cnl2^Nkp2^ and Mal2^CENP-O/Mcm21^, components of the constitutive CCAN/Mis6/Ctf19 complex. No Mis18BP1^KNL2^ orthologue has been identified in fission yeast, consequently it remains unknown how the key Cnp1^CENP-A^ loading factor Mis18 is recruited. Our findings suggest that Eic1 serves a function analogous to that of Mis18BP1^KNL2^, thus representing the functional counterpart of Mis18BP1^KNL2^ in fission yeast that connects with a module within the CCAN/Mis6/Ctf19 complex to allow the temporally regulated recruitment of the Mis18/Scm3^HJURP^ Cnp1^CENP-A^ loading factors. The novel interactions identified between CENP-A loading factors and the CCAN/Mis6/Ctf19 complex are likely to also contribute to CENP-A maintenance in other organisms.

## 2. Introduction

Genome integrity and stability are key to the propagation of genetic information through generations. Centromeres are the specialized sites on eukaryotic chromosomes where kinetochores, which govern spindle microtubule attachment, assemble during cell division. Centromere dysfunction, and consequent kinetochore instability, can therefore cause mis-segregation of chromosomes resulting in aneuploidy and genome instability. A plethora of evidence suggests that DNA sequence is neither necessary nor sufficient for the establishment of a functional centromere [1,2], and it is generally accepted that centromere specification is epigenetically regulated in many organisms [3,4].

CENP-A, a histone H3 variant, replaces canonical H3 in specialized nucleosomes that mark active centromeres. CENP-A is both necessary and sufficient to mediate the establishment and maintenance of functional kinetochores. CENP-A associates with a distinct set of proteins that allow it to promote its own propagation to preserve centromere location [5–7]. In the fission yeast *Schizosaccharomyces pombe*, Cnp1^CENP-A^ is restricted to the non-repetitive central domain of centromeres (approx. 7–10 kb; unique central core plus inverted inner-most repeats) that is flanked by heterochromatic outer repeats. Cnp1^CENP-A^ recruitment to the central domain depends on the conserved kinetochore proteins Mis18, Mis6^CENP-I/Ctf3^, Sim4^CENP-K/Mcm22^ and Scm3^HJURP^ [8–12]. Mis18 was originally identified as being required to maintain Cnp1^CENP-A^ at fission yeast centromeres. Subsequently, Mis18 function was shown to be conserved at vertebrate centromeres [11,13]. Mis18 associates with the nuclear protein Mis16, which is orthologous to *Saccharomyces cerevisiae* Hat2 and human RbAp46/48, which are known to act as histone chaperones in many distinct histone transactions [14,15]. Mis6^CENP-I/Ctf3^ and Sim4^CENP-K/Mcm22^ reside in a larger complex consisting of approximately 16–18 centromeric proteins [16–18], most of which are conserved and collectively form the complex known as CCAN, Ctf19 and Mis6 in vertebrates, budding yeast and fission yeast, respectively [16,19,20]. The modular CCAN/Mis6/Ctf19 complex forms the main scaffold of centromere proteins that is required to recruit outer kinetochore components in order to mediate and regulate kinetochore–microtubule attachments [21]. Scm3^HJURP^ is orthologous to the vertebrate CENP-A-specific chaperone HJURP [22]. Scm3^HJURP^ directly associates with Cnp1^CENP-A^ and is required for Cnp1^CENP-A^ incorporation at centromeres [9,12].

In vertebrate cells, the assembly of new CENP-A into centromeric chromatin is uncoupled from replication and occurs in early G1, prior to S phase [23]. Consequently, following centromere replication, the level of resident CENP-A at centromeres is halved as a result of its distribution to the resulting two sister-centromeres [24]. The CENP-A loading factors Mis18α/β and Mis18BP1^KNL2^ and the CENP-A chaperone HJURP^Scm3^ are transiently recruited to human centromeres in telophase and are required to replenish CENP-A in G1 [13,25,26]. Mis18 is recruited to human centromeres by Mis18BP1^KNL2^, which associates with centromeres via the C-terminus of the constitutive kinetochore component, CENP-C^Cnp3/Mif2^ [27,28]. Both Mis18α and Mis18BP1^KNL2^ are required for the cell-cycle-regulated recruitment of HJURP^Scm3^ prior to G1 [25,28,29]. Interestingly, in fission yeast, the CENP-C orthologue Cnp3 is not essential for viability [30] and no Mis18BP1^KNL2^ protein can be identified. It therefore remains unclear how Mis16^RbAp46/48/Hat2^, Mis18 and Scm3^HJURP^ are recruited to centromeres to maintain Cnp1^CENP-A^ at fission yeast centromeres. Nevertheless, as in vertebrate cells, the Cnp1^CENP-A^ assembly factors Mis16^RbAp46/48/Hat2^, Mis18 and Scm3^HJURP^ all display dynamic association with fission yeast centromeres during the cell cycle, but they are released from centromeres in mitotic prophase and re-associate in mid-anaphase [9,11–13]. This suggests that fission yeast relies on an alternative to the CENP-C^Cnp3/Mif2^–Mis18BP1^KNL2^ interaction for the regulated recruitment of the Mis16^RbAp46/48/Hat2^, Mis18 and Scm3^HJURP^ Cnp1^CENP-A^ assembly factors to centromeres.

Although many kinetochore proteins have clearly been conserved between different experimental organisms over evolutionary time, it is also evident that particular proteins have been lost in some organisms and replaced by alternative pathways. For example, both *Drosophila* and *S. cerevisiae* lack the Mis18 and Mis18BP1^KNL2^ proteins. However, *S. cerevisiae* retains an Scm3^HJURP^ orthologue, whereas Scm3^HJURP^ function is replaced by the distinct Cal1 protein in *Drosophila* [31,32]. Moreover, in *Arabidopsis* the association dynamics of Mis18BP1^KNL2^ with centromeres is more similar to that of *S. pombe* Mis18/Scm3^HJURP^ than human Mis18BP1^KNL2^/Mis18/HJURP^Scm3^ in that it departs only briefly from centromeres during mitosis, re-associating in mid-anaphase prior to *Arabidopsis* CenH3^CENP-A^ deposition in G2 [33,34]. It is well recognized that alternative solutions can evolve to mediate the same conserved process, and such alternatives can inform on parallel or other underlying pathways and mechanisms that may be more prevalent in one system relative to another.

In fission yeast, Mis16^RbAp46/48/Hat2^–Mis18 may promote Cnp1^CENP-A^ assembly through a direct physical interaction detected *in vitro* with the Cnp1^CENP-A^ chaperone Scm3^HJURP^ [9,12]. However, as no direct interaction has been reported between Mis16^RbAp46/48/Hat2^–Mis18 and constitutive CCAN components, it remains unknown how the Mis16^RbAp46/48/Hat2^–Mis18 and Scm3^HJURP^ assembly factors are recruited to the underlying constitutive kinetochore scaffold. Here, we set out to determine how fission yeast solves the problem of recruiting the Cnp1^CENP-A^ loading machinery (Mis16^RbAp46/48/Hat2^/Mis18/Scm3^HJURP^) to centromeres without a Mis18BP1^KNL2^ orthologue. Through a proteomics approach, we identified two previously uncharacterized proteins as additional integral components of the Mis16^RbAp46/48/Hat2^-Mis18 complex, designated Eic1 and Eic2 (Eighteen Interacting Centromere proteins 1 and 2). Our analyses indicate that Eic1 is essential to maintain normal Cnp1^CENP-A^ levels at centromeres, whereas Eic2 is dispensable. Importantly, we also demonstrate that Eic1 provides a link between Mis16^RbAp46/48/Hat2^-Mis18 and specific constitutive components of the kinetochore scaffold (CCAN/Mis6/Ctf19 complex components). The analyses presented identify three components within the conserved CCAN/Mis6/Ctf19 complex which we propose represent a module that is required to recruit the Cnp1^CENP-A^ loading machinery (Mis16^RbAp46/48/Hat2^/Mis18/Eic1/Scm3^HJURP^) to fission yeast centromeres.

## 3. Results

### 3.1. Identification of novel proteins that associate with Mis16^RbAp46/48/Hat2^ or Mis18

**Figure 1.**
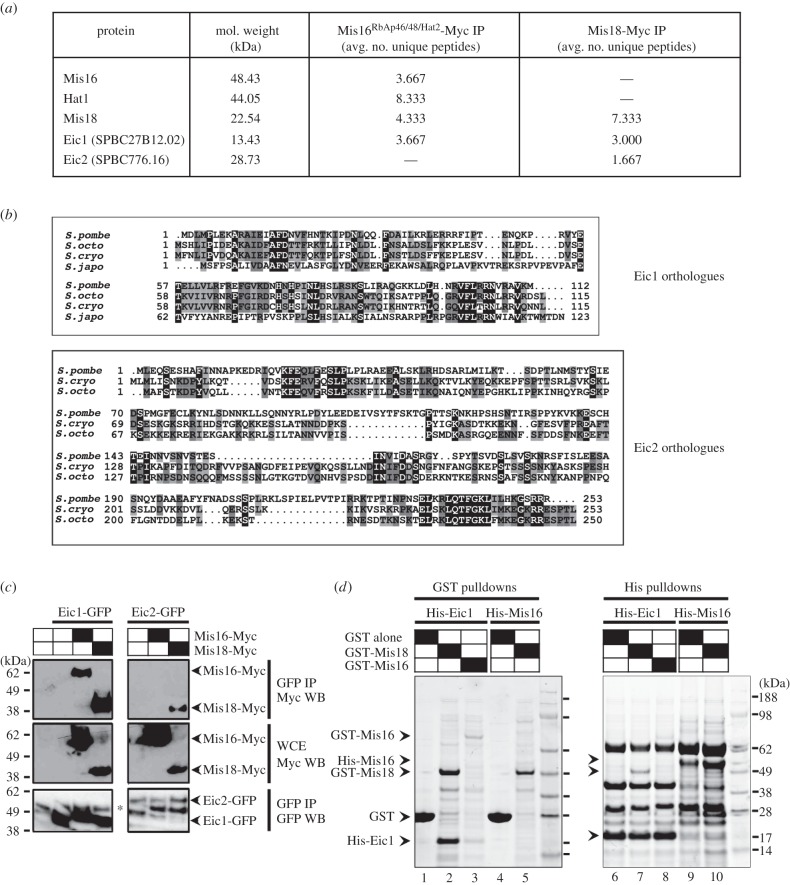
Eic1 and Eic2 are Mis18-interacting proteins. (*a*) LC-MS/MS analysis of Myc-tagged Mis16^RbAp46/48/Hat2^ and Mis18 immunoprecipitates from *S. pombe* whole cell extracts identifies two previously uncharacterized proteins, Eic1 (SPBC27B12.02) and Eic2 (SPBC776.16). Average number of unique peptides reproducibly identified from three independent experiments is shown. (*b*) Primary sequence alignments of Eic1 (top) and Eic2 (bottom) orthologues identified among *Schizosaccharomyces* species. (*c*) Eic1-GFP co-immunoprecipitates with both Mis16-Myc and Mis18-Myc, while Eic2-GFP only co-immunoprecipitates with Mis18-Myc. The asterisk (*) in the bottom panel denotes the IgG heavy chain. (*d*) Eic1 directly interacts with Mis18 and Mis16. 6xHis-Eic1 was co-expressed with GST alone, GST-Mis18 or GST-Mis16 in *E. coli*. Coomassie-stained SDS-PAGE gels showing reciprocal GST (lanes 1–3) and His (lanes 6–8) pulldowns from *E. coli* lysates are shown. Also shown are reciprocal pulldowns of 6xHis-Mis16 co-expressed with GST alone or GST-Mis18 (lanes 4–5 and 9–10).

To explore the interactions of Mis16^RbAp46/48/Hat2^ and Mis18 with other proteins in fission yeast, Myc-tagged Mis16 and Mis18 were immunoprecipitated from extracts of cells expressing either Mis16-Myc or Mis18-Myc as fusion proteins from the endogenous genes and the captured proteins subjected to LC-MS/MS analyses. Mis18 immunoprecipitates were reproducibly found to contain two previously uncharacterized proteins, which we named Eic1 (SPBC27B12.02) and Eic2 (SPBC776.16) for Eighteen Interacting Centromere protein 1 and 2, respectively. Mis16^RbAp46/48/Hat2^ immunoprecipitates also reproducibly contained Eic1, along with two other known interactors: Mis18 and the histone H4 acetyltransferase Hat1 (figure 1*a*) [11,35]. Proteins orthologous to Eic1 were identifiable in all the sequenced genomes of the three other fission yeast species (*S. octosporus*, *S. cryophilus* and *S. japonicus*) by homology and synteny searches (figure 1*b*). However, no orthologue of Eic2 was apparent in *S. japonicus* (figure 1*b*).

Co-immunoprecipitation experiments verified the interactions of Eic1 and Eic2 with Mis16^RbAp46/48/Hat2^ and Mis18. Consistent with the LC-MS/MS results, both Mis16-Myc and Mis18-Myc were found to co-immunoprecipitate with Eic1-GFP. Also, Mis18-Myc, but not Mis16-Myc, co-immunoprecipitated with Eic2-GFP (figure 1*c*). This demonstrates that Mis16^RbAp46/48/Hat2^, Mis18, Eic1 and Eic2 associate.

To determine whether Eic1 can directly associate with Mis16^RbAp46/48/Hat2^ or Mis18, we co-expressed 6xHis-Eic1 with GST-Mis16 or GST-Mis18 in *Escherichia coli*. GST-Mis18 and 6xHis-Eic1 can clearly associate with each other in reciprocal pull down assays (figure 1*d*, lanes 2 and 7). 6xHis-Eic1 could also be detected in pulldowns of GST-Mis16 (figure 1*d*, lane 3). Similarly, a weak interaction between 6xHis-Mis16 and GST-Mis18 could be detected *in vitro* (figure 1*d*, lane 5 when compared with lanes 4 or 2).

### 3.2. Hat1 does not contribute to the maintenance of Cnp1^CENP-A^ chromatin

In *S. cerevisiae*, Hat2 is the orthologue of Mis16 and it has previously been shown to associate with the histone H4 acetyl-transferase Hat1 [36]. Related to this, levels of acetylated histones have been shown to increase at centromeres in *mis16* and *mis18* mutants [11]. It is possible that altered histone acetylation at centromeres relates to the function of Mis16^RbAp46/48/Hat2^ and Hat1 at centromeres. We first confirmed that Mis16-Myc and Hat1-HA co-immunoprecipitate (electronic supplementary material, figure S1*a*) and then, to investigate the possibility that Hat1 possesses centromere-specific functions, we subjected Hat1-HA immunoprecipitates to LC/MS-MS analyses. However, we were unable to detect any Hat1-associated proteins apart from Mis16^RbAp46/48/Hat2^, confirming the analyses of others [35]. Moreover, Hat1-HA was not found to be enriched in the central kinetochore domain of centromeres (electronic supplementary material, figure S1*b*) and no loss of Cnp1^CENP-A^ from centromeres could be detected in cells lacking Hat1 (*hat1*Δ; electronic supplementary material, figure S1*c*). Thus, it appears that Mis16^RbAp46/48/Hat2^ may participate in two distinct functional complexes: Mis16^RbAp46/48/Hat2^–Mis18–Eic1–Eic2 at centromeres (see below) and Mis16^RbAp46/48/Hat2^–Hat1 elsewhere in the nucleus (electronic supplementary material, figure S1*d*). Such a multi-functional role for Mis16^RbAp46/48/Hat2^ would be consistent with the known involvement of RbAp46/48 proteins in many complexes involved in histone modification and chromatin remodelling [14,15].

### 3.3. Eic1 and Eic2 associate with centromeres dynamically through the cell cycle

**Figure 2.**
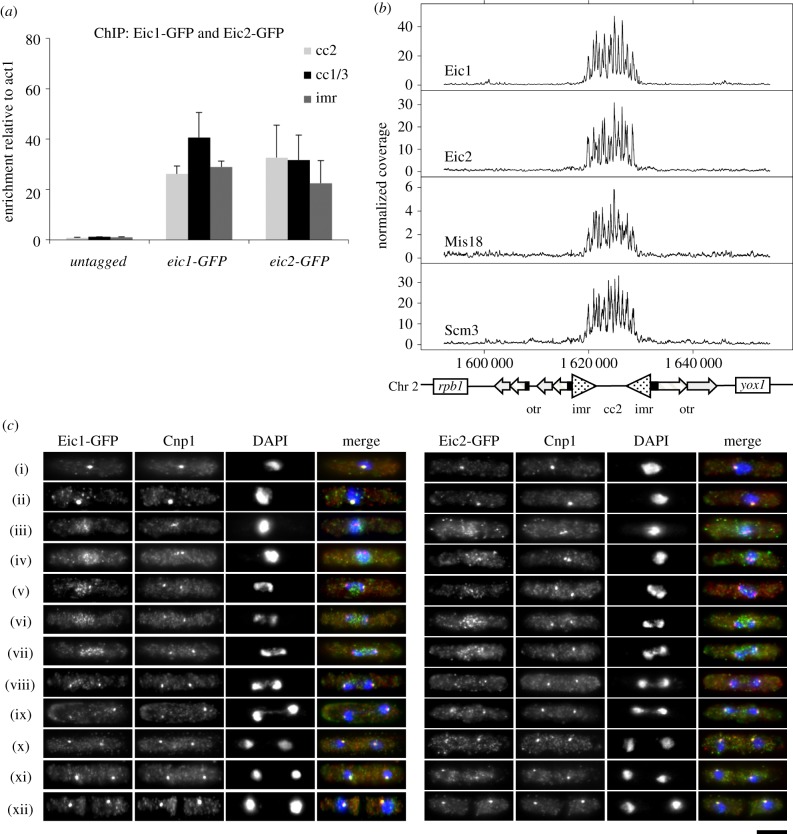
Eic1 and Eic2 associate specifically with centromeres. (*a*) Eic1 and Eic2 bind the central domain of *S. pombe* centromeres. qChIP analyses showing enrichments of GFP-tagged Eic1 and Eic2 at the central cores (cc) of centromeres 1, 2 and 3 and *imr* repeats of centromere 1, relative to the *act1* locus. Error bars represent standard deviation between at least three biological replicates. (*b*) Eic1 and Eic2 exhibit very similar genome-wide association profiles as Mis18 and Scm3^HJURP^. A comparison between the ChIP-seq profiles of GFP-tagged Eic1, Eic2, Mis18 and Scm3 across centromere 2 is presented, alongside a schematic diagram of centromere 2 (bottom). Normalized coverage represents the number of sequencing fragments obtained from anti-GFP IP normalized to that obtained from the input. (*c*) Eic1 and Eic2 exhibit very similar cell-cycle localization dynamics as Mis18 and Scm3^HJURP^. Immunofluorescence of *S. pombe* cells expressing GFP-tagged Eic1 or Eic2 stained with antibodies to GFP (green) and Cnp1^CENP-A^ (red), and DAPI (blue). Both Eic1 and Eic2 dissociate from centromeres during prometaphase to mid-anaphase of mitosis ((iii)–(vii)) and subsequently reassociate. Scale bar, 5 μm.

CENP-A and all known fission yeast kinetochore proteins localize specifically at centromeres and are enriched over the central domain region of centromeres. Both Eic1 and Eic2 associate with Mis18, which is known to localize to fission yeast centromeres for most of the cell cycle, apart from early prophase to mid-anaphase of mitosis [13]. To examine the localization of Eic1 and Eic2, the endogenous genes expressing Eic1 and Eic2 were fused to GFP. Quantitative chromatin immunoprecipitation (qChIP) analyses demonstrated that, as with Mis18 and other kinetochore proteins, both Eic1-GFP and Eic2-GFP are enriched over the central kinetochore domain (central core plus imr repeats) of fission yeast centromeres (figure 2*a*). Genome-wide ChIP-seq analyses confirmed this finding and also demonstrated that Eic1-GFP and Eic2-GFP, much like Mis18-GFP and Scm3-GFP, are undetectable at other regions of the genome including the heterochromatic outer repeats of centromeres (figure 2*b* and electronic supplementary material, figure S2). Immunostaining revealed that both Eic1 and Eic2 GFP-tagged proteins co-localize with Cnp1^CENP-A^ at clustered centromeres in interphase cells (figure 2*c*(i),(ii)). However, as with Mis16^RbAp46/48/Hat2^ and Mis18 [11,13], Eic1-GFP and Eic2-GFP dissociate from centromeres in early mitosis (prometaphase), just as the centromeres (Cnp1) begin to form two separate clusters as they biorient on the spindle (figure 2*c*(iii),(iv)). Moreover, both Eic1-GFP and Eic2-GFP reassociate with centromeres in mid-anaphase when centromeres (Cnp1) and chromosomes (DAPI) have clearly segregated to opposite spindle poles (figure 2*c*(viii),(ix)). Apart from Mis16^RbAp46/48/Hat2^ and Mis18, the CENP-A chaperone Scm3^HJURP^ exhibits similar temporal dissociation–reassociation at centromeres during mitosis [9,12]. Thus, our analyses identify Eic1 and Eic2 as two additional proteins that are released from centromeres in early mitosis and reloaded on centromeres in mid-anaphase. The finding that Eic1 and Eic2 exhibit similar association dynamics as Mis18 through the cell cycle is consistent with our identification of Eic1 and Eic2 being tightly associated with Mis18.

### 3.4. Eic1 is required to maintain Cnp1^CENP-A^ at centromeres

**Figure 3.**
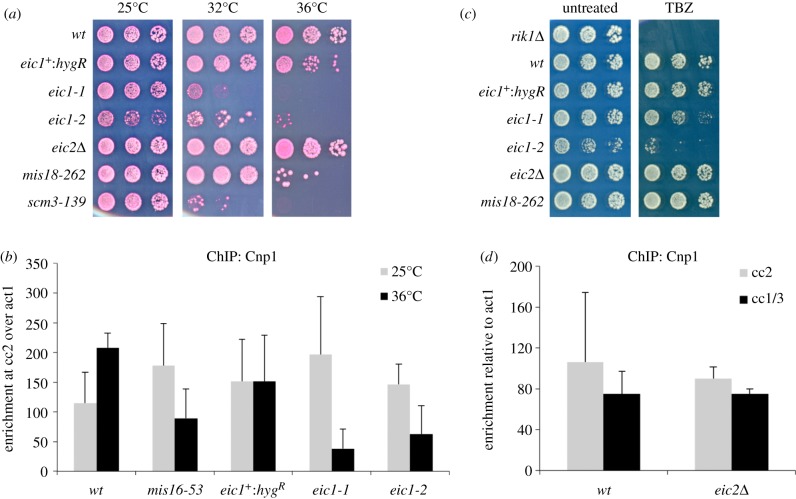
Eic1 is required for Cnp1^CENP-A^ assembly, while Eic2 is dispensable. (*a*) *ts* mutations in *eic1* affect cell viability, while *eic2*Δ cells show no defects in growth. Five-fold serial dilutions of cells spotted on YES + Phloxine B media and incubated at the indicated temperatures; dead cells stain dark pink. (*b*) *eic1* mutants display reduced Cnp1^CENP-A^ levels at centromeres. qChIP analyses of Cnp1^CENP-A^ association with centromeres in the indicated strains when grown at permissive (25°C) versus restrictive temperature (36°C) for 8 h. Enrichment of cc2 DNA relative to the *act1* locus is presented. (*c*) *eic1* mutants display sensitivity to TBZ, while *eic2*Δ cells show no TBZ sensitivity. Five-fold serial dilutions of cells spotted on YES media (untreated) or YES media supplemented with 12.5 μg ml^−1^ TBZ, and incubated at 25°C. (*d*) *eic2*Δ cells display no loss of Cnp1^CENP-A^ at centromeres. qChIP analyses of Cnp1^CENP-A^ association with centromeres in the indicated strains when grown at 32°C. Enrichment of cc2 or cc1/3 DNA relative to the *act1* locus is presented. Error bars in (*b*,*d*) represent standard deviation between at least three biological replicates.

Mis18 and associated proteins have been shown to be required to maintain CENP-A at centromeres in fission yeast and vertebrates [11,13,37]. If Eic1 and Eic2 are critical for Mis18 function then they should also be required to maintain normal Cnp1^CENP-A^ levels at centromeres. As the *eic1*^+^ gene is essential for cell viability, we generated a conditional temperature-sensitive (*ts*) mutant of *eic1* (*eic1*-*1*:*hygR*, hereafter referred to as *eic1-1*) in which a single amino acid substitution (Phe102Ser) at a conserved residue rendered cells inviable at 36°C but with retained viability at 25°C (figure 3*a*). We also generated a corresponding wild-type allele of *eic1* (*eic1*^+^:*hyg^R^*) as a control in which, as with *eic1-1*, a hygromycin resistance marker was inserted within the 3′UTR of *eic1* at its endogenous locus for ease of genetic manipulation (figure 3*a*). qChIP analyses demonstrated that Cnp1^CENP-A^ levels were significantly diminished at centromeres in *eic1-1* cells at restrictive temperature (figure 3*b*; electronic supplementary material, figure S3*a*). Cells harbouring a second *ts* allele of *eic1* (*eic1-2*:*hyg*^*R*^; two substituted residues, Lys37Glu and Tyr55His, hereafter referred to as *eic1-2*) also demonstrated significant loss of Cnp1^CENP-A^ from centromeres at 36°C (figure 3*a*,*b*; electronic supplementary material, figure S3*a*). Furthermore, *eic1-1* and *eic1-2* cells displayed sensitivity to the microtubule depolymerizing drug thiabendazole (TBZ), whereas *eic1^+^:hyg^R^* and the *mis18-262* ts mutant did not confer TBZ sensitivity (figure 3*c*). TBZ sensitivity suggests that kinetochore–microtubule interactions are defective in *eic1* mutants. Aberrant kinetochore function in *eic1-1* cells was supported by cytological analyses, which revealed severe chromosome segregation defects (electronic supplementary material, figure S3*b*). qChIP analyses demonstrated that GFP-tagged Eic1-1 and Eic1-2 mutant proteins remained associated with centromeres even at 36°C; thus, the observed phenotypes of *eic1-1* and *eic1-2* cells are not a consequence of the complete absence of Eic1 protein at centromeres (electronic supplementary material, figure S3*c*).

In contrast to *eic1* mutants, cells lacking the *eic2^+^* gene (*eic2*Δ) displayed no loss of Cnp1^CENP-A^ from centromeres (figure 3*d*). One possibility is that Eic2 is not required to maintain Cnp1^CENP-A^ at centromeres but is required to establish Cnp1^CENP-A^ on naive centromeric DNA. To test this, a plasmid bearing centromeric DNA that efficiently establishes Cnp1^CENP-A^ chromatin and functional kinetochores in wild-type cells (pH-cc2) [38] was transformed into *eic2*Δ cells. No defect in the establishment of Cnp1^CENP-A^ chromatin or functional kinetochores was observed (electronic supplementary material, figure S4). Furthermore, *eic2*Δ cells were not sensitive to TBZ (figure 3*c*). We conclude that Eic1 is required for Cnp1^CENP-A^ maintenance and kinetochore integrity, whereas Eic2 is dispensable.

### 3.5. The recruitment of Cnp1^CENP-A^ assembly factors is reduced at centromeres in *eic1* and *eic2* mutants

**Figure 4.**
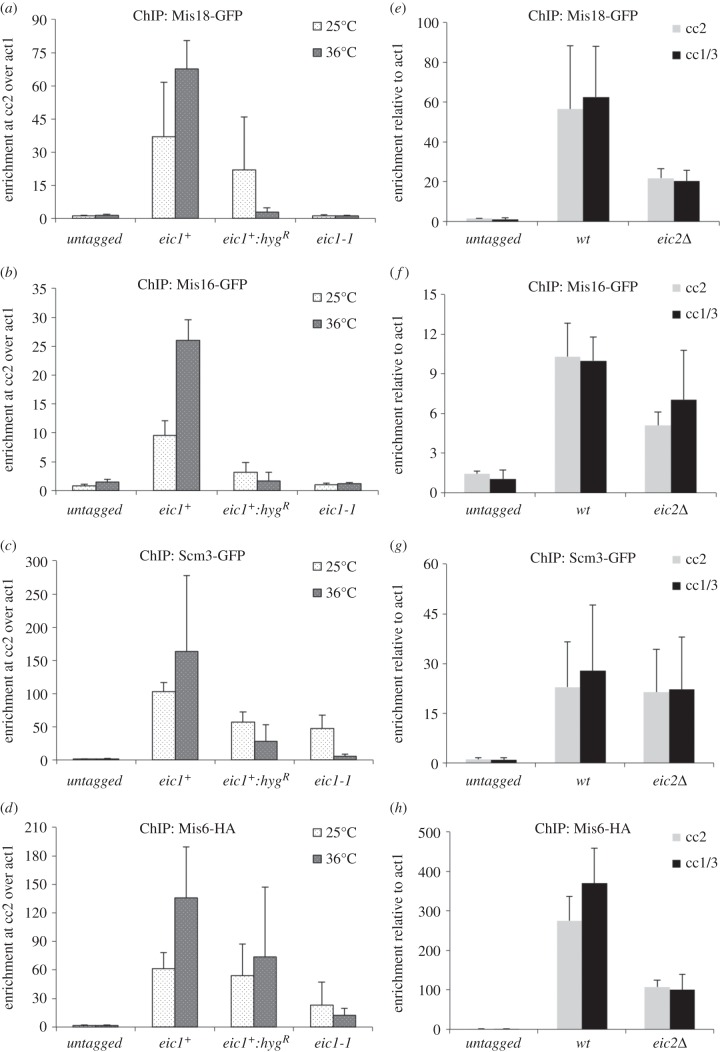
Eic1 and Eic2 promote normal levels of association of Cnp1^CENP-A^ assembly factors with centromeres. (*a*,*b*) Mis18-GFP and Mis16-GFP association with centromeres is entirely dependent on Eic1, and (*e*,*f*) partly dependent on Eic2. (*c*) Scm3-GFP association with centromeres is dependent on Eic1, and (*g*) largely independent of Eic2. (*d*) Mis6-HA association with centromeres is partly dependent on Eic1, and (*h*) Eic2. qChIP analyses of (*a*,*e*) Mis18-GFP, (*b*,*f*) Mis16-GFP, (*c*,*g*) Scm3-GFP and (*d*,*h*) Mis6-HA association with centromeres in the indicated strains when grown at permissive (25°C) versus restrictive temperature (36°C) for 8 h (*a*–*d*); or at 32°C (*e*–*h*). Enrichment of cc2 DNA relative to the *act1* locus is presented in (*a*–*d*). Enrichment of cc2 or cc1/3 DNA relative to the *act1* locus is presented in (*e*–*h*). Error bars represent standard deviation between at least three biological replicates.

Previous studies have shown that defective Mis16^RbAp46/48/Hat2^ or Mis18 function affects the localization of the Cnp1^CENP-A^ loading factors Scm3^HJURP^ and Mis6^CENP-I/Ctf3^ at centromeres [9,11,12]. Vertebrate Mis18α/β and Mis18BP1^KNL2^ have also been shown to be required for the recruitment of the CENP-A chaperone HJURP^Scm3^ to centromeres, and consequently the incorporation of CENP-A at centromeres [13,25]. We find that the levels of GFP-tagged Mis16^RbAp46/48/Hat2^ and Mis18 associated with fission yeast centromeres are greatly reduced in the *eic1-1* mutant even at 25°C (figure 4*a*,*b*), consistent with the observed synthetic genetic interaction between *eic1-1* and the GFP-tagged alleles of *mis16* and *mis18* (electronic supplementary material, figure S5). Additionally, we find that Scm3^HJURP^ association with centromeres is also dependent on Eic1 function (figure 4*c*). Interestingly, we find Mis6^CENP-I/Ctf3^ association with centromeres to be only partially dependent on Eic1 (figure 4*d*), as intermediate levels of Mis6^CENP-I/Ctf3^ are retained at centromeres even when Eic1 function is compromised. Thus Eic1, in conjunction with its interacting partners Mis16^RbAp46/48/Hat2^ and Mis18, is essential to maintain normal levels of Cnp1^CENP-A^ as well as Cnp1^CENP-A^ loading factors on centromeres.

Surprisingly, *eic2*Δ cells exhibited reduced levels of Mis18, Mis16^RbAp46/48/Hat2^ and Mis6^CENP-I/Ctf3^ at centromeres, whereas Scm3^HJURP^ remained unaffected (figure 4*e*–*h*). As normal levels of Cnp1^CENP-A^ are retained at centromeres and no defect in chromosome segregation was detectable in *eic2*Δ cells (figure 3*c*,*d* and electronic supplementary material, figure S4), the observed reduction of Mis18, Mis16^RbAp46/48/Hat2^ and Mis6^CENP-I/Ctf3^ at centromeres must not be sufficient to significantly affect Cnp1^CENP-A^ maintenance.

**Figure 5.**
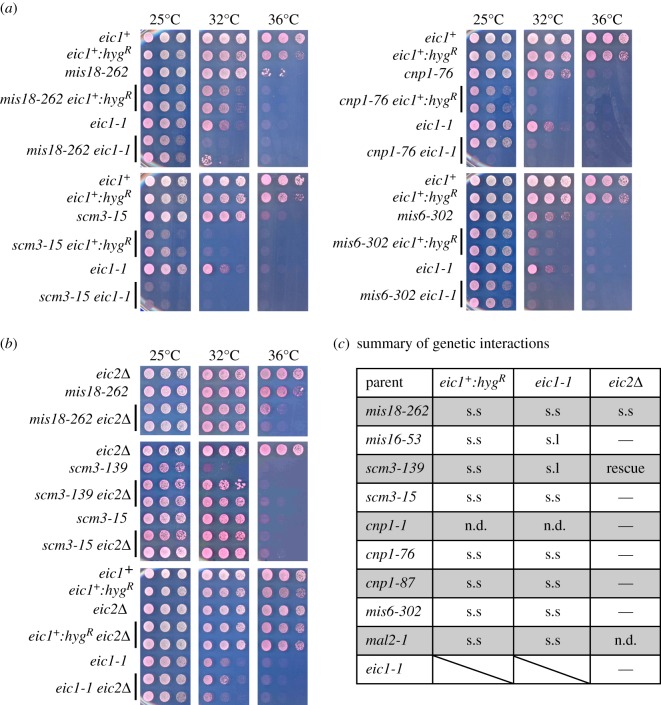
Analysis of genetic interactions between *eic1* or *eic2* mutants and mutations in Cnp1^CENP-A^ or Cnp1^CENP-A^ assembly factors. (*a*) *eic1^+^:hyg^R^* and *eic1-1* cells display reduced growth when combined with mutations in *mis18*, *scm3*, *cnp1* or *mis6*. (*b*) *eic2*Δ cells display genetic interactions when combined with *mis18-262* and *scm3-139*, but not *eic1-1*. Five-fold serial dilutions of cells spotted on YES + Phloxine B media and incubated at the indicated temperatures; dead cells stain dark pink. (*c*) A tabular summary of genetic interactions analysed in this study. s.s, synthetic sick/reduced growth; s.l, synthetic lethal; n.d., not determined; —, no interaction detected.

Epistasis analyses (double mutant combinations) frequently reveal positive and negative genetic interactions between mutants and consequently inform on the functional niche of specific proteins. The *eic1-1* mutation exhibited significantly reduced growth when combined with *ts* mutations in *mis18*, *scm3*, *cnp1* and *mis6* (figure 5*a* and electronic supplementary material, figure S6*a*; summarized in figure 5*c*). We failed to generate *eic1-1 mis16-53* and *eic1-1 scm3-139* double mutant strains, suggesting that *eic1-1* has a synthetic lethal interaction with both *mis16-53* and *scm3-139*. Surprisingly, such synthetic interactions could also be observed for the *eic1^+^:hyg^R^* allele, which must compromise *eic1* function in sensitized genetic backgrounds and therefore be considered a hypomorphic allele of *eic1* (figure 5 and electronic supplementary material, figure S6*a*). These results, in conjunction with our ChIP analyses of Cnp1^CENP-A^ assembly factors (figure 4*a*–*d*), clearly demonstrate that Eic1 makes important contributions to the proper localization of Cnp1^CENP-A^ assembly factors and Cnp1^CENP-A^ itself.

*eic2*Δ showed no combinatorial effects when combined with *cnp1*, *mis6* or *mis16* *ts* mutants (electronic supplementary material, figure S6*b*; summarized in figure 5*c*). However, *eic2*Δ displayed a negative interaction with *mis18-262*, notably reducing growth at 36°C relative to *mis18-262* alone (figure 5*b*). By contrast, *eic2*Δ partially suppressed the temperature sensitivity of the *scm3-139* (Leu73Phe) but not the *scm3-15* (Ser281Leu) mutant (figure 5*b*; summarized in figure 5*c*). The negative interaction of Eic2 with Mis18 confirms that Eic2 contributes to the function of the Mis18 complex. As the protein levels as well as association of Scm3^HJURP^ with centromeres remain unaffected in *eic2*Δ cells (figure 4*c*; electronic supplementary material, figure S6*c*), the mechanism of partial suppression of temperature sensitivity observed in *eic2*Δ *scm3-139* cells remains unclear. *eic2*Δ showed no combinatorial effects when combined with *eic1-1* (figure 5*b*; summarized in figure 5*c*), suggesting that Eic2 may not influence Eic1 function even though Eic1 and Eic2 physically associate.

### 3.6. Eic1 and Eic2 association with centromeres is dependent on Cnp1^CENP-A^ loading factors

**Figure 6.**
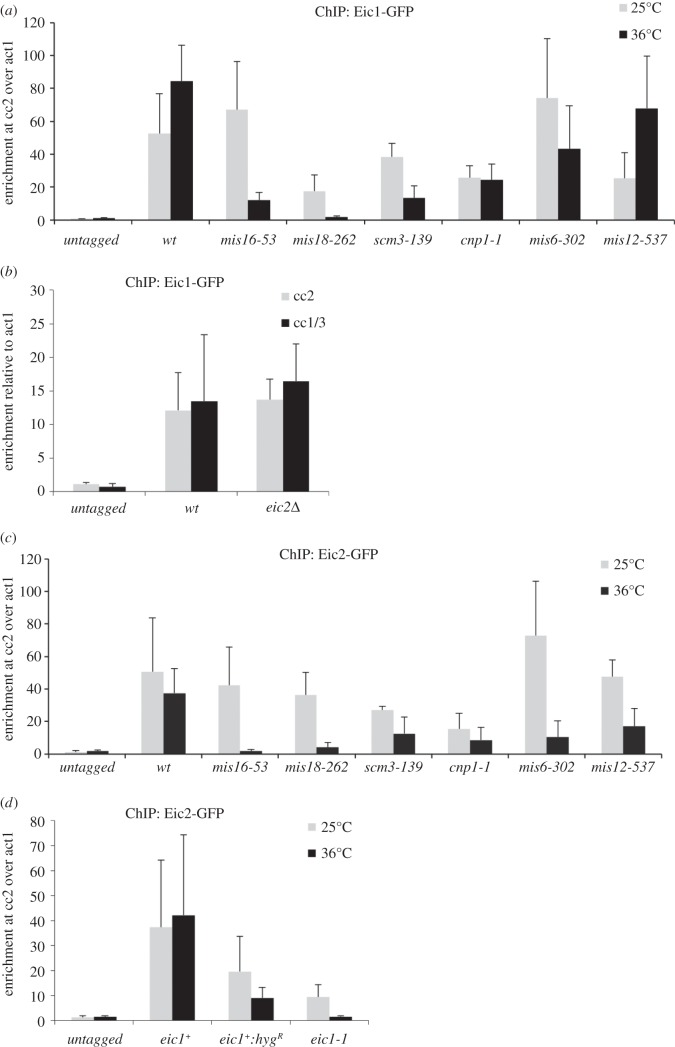
Eic1 and Eic2 depend on distinct Cnp1^CENP-A^ assembly factors for their association with centromeres. (*a*,*b*) Eic1 association with centromeres is dependent on Mis18, Mis16^RbAp46/48/Hat2^ and Scm3^HJURP^, but is largely independent of Cnp1^CENP-A^, Mis6^CENP-I/Ctf3^, Mis12 and Eic2. qChIP analyses of Eic1-GFP association with centromeres in the indicated strains when grown at permissive (25°C) versus restrictive temperature (36°C) for 8 h (*a*), or when grown at 32°C (*b*). (*c*,*d*) Eic2 association with centromeres is dependent on Mis18, Mis16^RbAp46/48/Hat2^, Scm3^HJURP^, Cnp1^CENP-A^, Mis6^CENP-I/Ctf3^, Mis12 and Eic1. qChIP analyses of Eic2-GFP association with centromeres in the indicated strains when grown at permissive (25°C) versus restrictive temperature (36°C) for 8 h. Enrichment of cc2 DNA relative to the *act1* locus is presented in (*a*,*c*,*d*). Enrichment of cc2 or cc1/3 DNA relative to the *act1* locus is presented in (*b*). Error bars represent standard deviation between at least three biological replicates.

Previous analyses in fission yeast have shown that Mis16^RbAp46/48/Hat2^ and Mis18 are required for the localization of the Cnp1^CENP-A^-specific chaperone Scm3^HJURP^ and the CCAN protein Mis6^CENP-I/Ctf3^ to centromeres [9,11,12]. Moreover, the localization of Mis18 at centromeres is unaffected by mutations in Cnp1^CENP-A^, Mis6^CENP-I/Ctf3^ or Scm3^HJURP^, whereas Scm3^HJURP^ is dependent on functional Mis6^CENP-I/Ctf3^, Mis16^RbAp46/48/Hat2^ and Mis18 for its centromeric localization [9,11,12]. Such analyses suggest that Mis18, and probably Mis16^RbAp46/48/Hat2^, function to mediate the recruitment of the Cnp1^CENP-A^ assembly factors Mis6^CENP-I/Ctf3^ and Scm3^HJURP^ to centromeres and thus the incorporation of Cnp1^CENP-A^ itself. To further dissect the role of Eic1 and Eic2, and to determine whether they act together with Mis16^RbAp46/48/Hat2^ and Mis18, we used qChIP to examine the dependencies of Eic1 and Eic2 for centromere localization in strains expressing various *ts* kinetochore proteins. Eic1 association with centromeres was found to be entirely dependent on functional Mis18, as indicated by undetectable levels of Eic1 at centromeres in *mis18-262* cells grown at 36°C (figure 6*a* and electronic supplementary material, figure S7*a*). Lower Eic1 levels were also detected on centromeric central cores in *mis16-53* and *scm3-139* mutants at restrictive temperature; however, they were largely unchanged in cells expressing mutant Cnp1^CENP-A^, Mis6^CENP-I/Ctf3^ and Mis12 proteins (figure 6*a* and electronic supplementary material, figure S7*a*). Centromeric Eic1 levels also remained unaffected in *eic2*Δ cells (figure 6*b*). Thus, the localization of Eic1 at kinetochores is mainly dependent on its partner proteins Mis16^RbAp46/48/Hat2^ and Mis18, but not Eic2.

The level of Eic2 at centromeres was found to be greatly dependent on functional Mis16^RbAp46/48/Hat2^, Mis18 and Eic1, and partially dependent on functional Scm3^HJURP^, Cnp1^CENP-A^ and Mis6^CENP-I/Ctf3^ (figure 6*c*,*d* and electronic supplementary material, figure S7*b*). Unexpectedly, Eic2 levels at centromeres were found to be reduced in *mis12-537* (a mutation in an outer kinetochore component) cells at restrictive temperature (figure 6*c* and electronic supplementary material, figure S7*b*). These analyses suggest that Eic2 is also recruited to centromeres by other mechanisms that are independent of its association with Mis18.

### 3.7. Eic1 associates with CCAN/Mis6/Ctf19 complex components that influence its recruitment to centromeres

**Figure 7.**
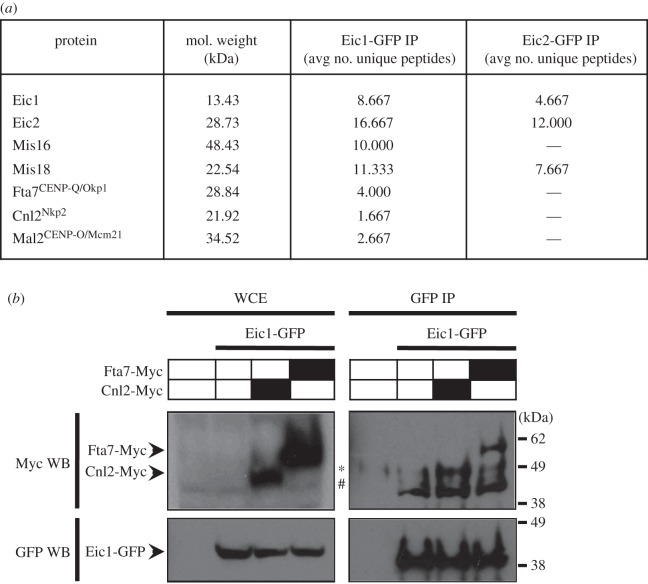
Eic1 interacts with three essential subunits of the CCAN/Mis6/Ctf19 complex. (*a*) LC-MS/MS analysis of GFP-tagged Eic1 or Eic2 immunoprecipitates from *S. pombe* whole cell extracts identifies Fta7^CENP-Q/Okp1^, Cnl2^Nkp2^ and Mal2^CENP-O/Mcm21^ as Eic1-interacting proteins. Average number of unique peptides reproducibly identified from three independent experiments is shown. (*b*) Eic1-GFP co-immunoprecipitates with Fta7-Myc and Cnl2-Myc. In the top panel, the asterisk (*) denotes the IgG heavy chain, and the hash tag (#) denotes a non-specific band.

**Figure 8.**
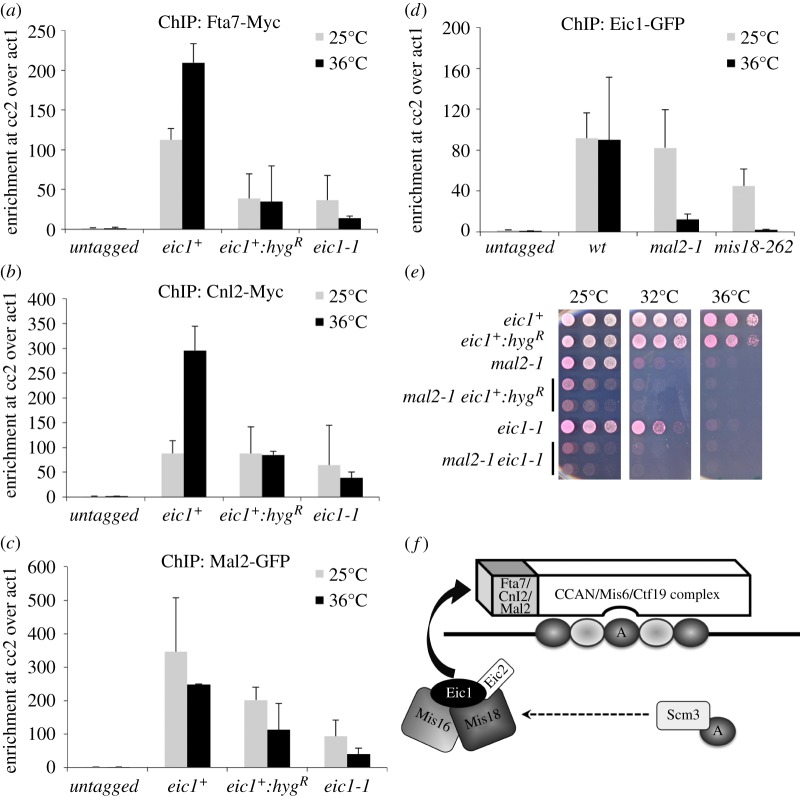
The CCAN components Fta7^CENP-Q/Okp1^, Cnl2^Nkp2^ and Mal2^CENP-O/Mcm21^ influence Eic1 association with centromeres. (*a*) Fta7-Myc, (*b*) Cnl2-Myc and (*c*) Mal2-GFP association with centromeres is partly dependent on Eic1. (*d*) Eic1-GFP association with centromeres is greatly dependent on Mal2. qChIP analyses of (*a*) Fta7-Myc, (*b*) Cnl2-Myc, (*c*) Mal2-GFP and (*d*) Eic1-GFP association with centromeres in the indicated strains when grown at permissive (25°C) versus restrictive temperature (36°C) for 8 h. Enrichment of cc2 DNA relative to the *act1* locus is presented. Error bars represent standard deviation between at least three biological replicates. (*e*) *eic1-1* displays a severe negative genetic interaction when combined with *mal2-1*. Five-fold serial dilutions of cells spotted on YES + Phloxine B media and incubated at the indicated temperatures; dead cells stain dark pink. (*f*) A model for Cnp1^CENP-A^ maintenance at *S. pombe* centromeres mediated by Eic1. The CCAN components Fta7^CENP-Q/Okp1^, Cnl2^Nkp2^ and Mal2^CENP-O/Mcm21^ that are constitutively bound to centromeres together form a module that recruits Eic1, and thereby regulates the temporal association of Eic1, Mis16^RbAp46/48/Hat2^, Mis18 and Eic2 with centromeres. Once bound, Mis16^RbAp46/48/Hat2^/Mis18 then likely recruit the Cnp1^CENP-A^-specific chaperone Scm3^HJURP^ to centromeres (the dashed arrow indicates that only an *in vitro* association between these proteins has been demonstrated), and thus ensures replenishment of Cnp1^CENP-A^ (‘A’ in closed oval) at centromeres in every cell cycle.

To further investigate the functional niche of Eic1 and Eic2, immunoprecipitates of both GFP-tagged proteins were subjected to LC-MS/MS analyses to identify associated factors. Only Eic1 and Mis18 were detected in Eic2-GFP immunoprecipitates (figure 7*a*). However, three CCAN/Mis6/Ctf19 complex components were found to associate with Eic1-GFP: Fta7^CENP-Q/Okp1^, Cnl2^Nkp2^ and Mal2^CENP-O/Mcm21^ (figure 7*a*). Co-immunoprecipitation of Myc-tagged Fta7^CENP-Q/Okp1^ or Cnl2^Nkp2^ with Eic1-GFP verified that these CCAN/Mis6/Ctf19 complex components associate with Eic1 (figure 7*b*). ChIP analyses revealed that the association of Myc-tagged Fta7^CENP-Q/Okp1^, Myc-tagged Cnl2^Nkp2^ and GFP-tagged Mal2^CENP-O/Mcm21^ with centromeres is only partially dependent on functional Eic1 (figure 8*a*–*c*), as these proteins are detectable to a significant degree at centromeres in *eic1-1* cells even at non-permissive temperature. This is consistent with the finding that centromeric association of the CCAN component Mis6^CENP-I/Ctf3^ also only partly depends on Eic1 function (figure 4*d*).

Interestingly, Eic1-GFP association with centromeres was found to greatly depend on functional Mal2^CENP-O/Mcm21^ (figure 8*d*), although it appeared to be largely independent of functional Mis6^CENP-I/Ctf3^ (figure 6*a* and electronic supplementary material, figure S7*a*). Additionally, the *mal2-1* ts mutant exhibited a more severe growth defect than *mis6-302* when combined with *eic1-1* (figures 5*a* and 8*e*). Together, these results suggest that the CCAN components Fta7^CENP-Q/Okp1^, Cnl2^Nkp2^ and Mal2^CENP-O/Mcm21^ may act in concert to recruit Eic1 to centromeres via their physical association with Eic1.

## 4. Discussion

In this study, we have identified and characterized two novel Mis18-interacting proteins, Eic1 and Eic2, in fission yeast. Eic1 is a small protein of approximately 13.4 kDa that is well conserved within *Schizosaccharomyces* species (figure 1*b*). The absence of an Eic2 orthologue in *S. japonicus* suggests that the primary sequence of Eic2 may have rapidly diverged over evolutionary time so that it is now undetectable within the *S. japonicus* genome; alternatively, other proteins may undertake the function of Eic2 in *S. japonicus*. The lack of any specific domains within Eic1 and Eic2 hinders the detection of similar or related proteins in other organisms, and thus orthologues of Eic1 and Eic2 remain unidentified outside of the *Schizosaccharomyces* clade.

The human Mis18 complex (consisting of hMis18α/β and Mis18BP1^KNL2^) primes CENP-A assembly by associating with centromeres specifically in telophase, subsequently recruiting the CENP-A-specific chaperone HJURP^Scm3^ to mediate CENP-A deposition in G1 [13,25,26,28,29]. The phosphorylation of Mis18BP1^KNL2^ by CDK1/2 has been shown to regulate the recruitment of Mis18BP1^KNL2^ and timing of CENP-A assembly during the cell cycle [39]. In fission yeast, the known Cnp1^CENP-A^ loading factors, Mis16^RbAp46/48/Hat2^, Mis18 and Scm3^HJURP^, also exhibit a very specific cell-cycle-regulated localization to centromeres in that they dissociate before metaphase and re-associate in mid-anaphase [9,11–13]. Our analyses demonstrate that both Eic1 and Eic2 are centromere-specific proteins with cell-cycle dynamics that are very similar to that of Mis16^RbAp46/48/Hat2^, Mis18 and Scm3^HJURP^ (figure 2 and electronic supplementary material, figure S2). The re-association of these five factors in anaphase–telophase is presumably required to allow the subsequent replication-independent deposition of Cnp1^CENP-A^ in G2 phase of the cell cycle [40,41]. Recent analysis of *Arabidopsis* Mis18BP1^KNL2^ suggests that it conforms to the *S. pombe*, rather than the vertebrate, pattern of CENP-A loading factor recruitment to centromeres during the cell cycle [33].

Our analyses show that functional Eic1 is required for the association of its interacting partners Mis16^RbAp46/48/Hat2^ and Mis18, as well as Scm3^HJURP^ with centromeres (figure 4*a*–*c*). The *eic1^+^:hyg^R^* and *eic1-1* alleles also display strong negative interactions when combined with mutant Cnp1^CENP-A^ or mutant Cnp1^CENP-A^ assembly factors, Mis16^RbAp46/48/Hat2^, Mis18 and Scm3^HJURP^ (figure 5*a*; summarized in figure 5*c*). Such interactions are consistent with Eic1 being required to promote Cnp1^CENP-A^ assembly by mediating the association of the Cnp1^CENP-A^ chaperone Scm3^HJURP^ as well as Mis16^RbAp46/48/Hat2^ and Mis18 with centromeres. Eic1 is required to recruit Mis18 to centromeres, and functional Mis18 has been shown to affect the localization of the CCAN/Mis6/Ctf19 complex component Mis6^CENP-I/Ctf3^ to centromeres [11]. It was thus surprising that our analyses only detected a partial reduction in Mis6^CENP-I/Ctf3^ levels at centromeres in *eic1* mutant cells (figure 4*d*). Nevertheless, consistent with this finding, our analyses show that the association of other CCAN components with centromeres is only partly dependent on Eic1 function (Fta7^CENP-Q/Okp1^, Cnl2^Nkp2^ and Mal2^CENP-O/Mcm21^; figure 8*a*–*c*). The CCAN proteins Mis6^CENP-I/Ctf3^, Sim4^CENP-K/Mcm22^, Mis15^CENP-N/Chl4^ and Mis17^CENP-U/Ame1^ have been shown to facilitate Cnp1^CENP-A^ assembly [8,10,11]. Thus, although functional Eic1 is essential for the recruitment of the key Cnp1^CENP-A^ assembly factors Mis16^RbAp46/48/Hat2^, Mis18 and Scm3^HJURP^ to centromeres, it is partly dispensable for maintaining constitutive CCAN components at centromeres.

Eic2, the second Mis18-interacting protein that we identified, contributes to Mis18 function (figure 5*b*) as it promotes normal levels of Mis18, Mis16^RbAp46/48/Hat2^ and Mis6^CENP-I/Ctf3^ association with centromeres (figure 4*e*,*f*,*h*). However, Eic2 is dispensable for maintaining Cnp1^CENP-A^ and Scm3^HJURP^ association with centromeres (figures 3*d* and 4*g*). It was therefore unexpected that loss of Eic2 (*eic2*Δ) resulted in the partial rescue of *scm3-139* temperature sensitivity (figure 5*b*; summarized in figure 5*c*). Given that the levels of Scm3^HJURP^ protein and its association with centromeres remain unaffected in *eic2*Δ cells (figure 4*g*; electronic supplementary material, figure S6*c*), we speculate that Scm3^HJURP^–Mis18 interactions are perhaps stabilized in the absence of Eic2. Regardless of these changes, the levels of loading factors remaining at centromeres in *eic2*Δ cells are sufficient to maintain normal Cnp1^CENP-A^ levels. The fact that the localization and function of Mis18, but not Eic1 or Cnp1^CENP-A^ (figures 3*d*, 4*e*, 5*b* and 6*b*), is compromised in *eic2*Δ cells suggests that Eic1 and Eic2 function independently. Eic2 may act to bolster Mis18 function under particular conditions of stress.

Eic1 association with centromeres is entirely dependent on functional Mis18, but only partly dependent on functional Mis16^RbAp46/48/Hat2^ and Scm3^HJURP^. Moreover, Eic1 levels at centromeres remain largely unaffected when Cnp1^CENP-A^, Mis6^CENP-I/Ctf3^ or Mis12 function is compromised (figure 6*a* and electronic supplementary material, figure S7*a*). These and other observations (figure 4*a*–*d*) suggest that Eic1 acts in concert with Mis16^RbAp46/48/Hat2^ and Mis18 to promote recruitment of the Cnp1^CENP-A^-specific chaperone Scm3^HJURP^ to centromeres and thereby facilitates Cnp1^CENP-A^ assembly. Thus, although no obvious protein similarity is evident between Eic1 and Mis18BP1^KNL2^, we propose that Eic1 serves as the functional counterpart of Mis18BP1^KNL2^ [13, 33,37].

The maintenance of epigenetically specified centromeres on chromosomes requires a feedback mechanism where, once established, constitutive centromere proteins themselves recruit the CENP-A assembly factors and thereby ensure their preservation. In vertebrates, the CENP-A assembly factors HJURP^Scm3^/Mis18/Mis18BP1^KNL2^ are recruited by the interaction of Mis18BP1^KNL2^ with CENP-C^Cnp3/Mif2^ [27,28]. Our analyses suggest that Eic1 performs the equivalent function to Mis18BP1^KNL2^ by association with the Fta7^CENP-Q/Okp1^, Cnl2^Nkp2^ and Mal2^CENP-O/Mcm21^ subunits of the constitutive CCAN/Mis6/Ctf19 complex (figure 7). In support of this view, we find that Eic1 recruitment to centromeres is particularly dependent on functional Mal2^CENP-O/Mcm21^, and *eic1 mal2* double mutants are severely compromised (figure 8*d*,*e*). By contrast, Eic1 association with centromeres is only partly dependent on the CCAN component Mis6^CENP-I/Ctf3^ (figure 6*a* and electronic supplementary material, figure S7*a*). Mcm21^Mal2/CENP-O^ and Okp1^Fta7/CENP-Q^ along with Ctf19^Fta2/CENP-P^ and Ame1^Mis17/CENP-U^ are known to form COMA, a biochemically distinct subcomplex within the larger Ctf19 complex in *S. cerevisiae* [19,42], much like the stable CENP-O/P/Q/U subcomplex described in vertebrate cells [43]. We propose that Mal2^CENP-O/Mcm21^, Fta7^CENP-Q/Okp1^ and Cnl2^Nkp2^ form an analogous module within the CCAN/Mis6/Ctf19 complex that ensures the propagation of Cnp1^CENP-A^ chromatin and kinetochores by recruiting Eic1 and consequently the Cnp1^CENP-A^ assembly factors Mis16^RbAp46/48/Hat2^, Mis18 and Scm3^HJURP^ to fission yeast centromeres.

Undoubtedly, the analyses of centromere–kinetochore architecture in distinct model systems have provided insights into the function of particular components. The lack of specific components in one organism reveals its reliance on alternative pathways. For example, *Drosophila* lack the kinetochore protein CENP-T^Cnp20/Cnn1^, and consequently their kinetochores are completely reliant on the KMN (Knl1–Mis12–Ndc80) pathway for attachment to microtubules [4,18]. In vertebrates, Mis18BP1^KNL2^ directly associates with CENP-C^Cnp3/Mif2^, allowing the maintenance and propagation of centromeric chromatin through the recruitment of Mis18, HJURP^Scm3^ and thus CENP-A [27,28]. Remarkably, the CENP-A^CID^ assembly pathway appears to be completely rewired during evolution of *Drosophila* because the main CENP-A loading factors HJURP^Scm3^, Mis18 and Mis18BP1^KNL2^ have been lost and replaced by the Cal1 protein, which directly associates with CENP-C^Cnp3/Mif2^ [31,32]. In *S. cerevisiae*, where centromere specification is driven by the recognition of specific DNA elements by DNA-binding proteins [15], a self-propagation mechanism is no longer necessary and consequently proteins equivalent to Mis18/Mis18BP1^KNL2^, Cal1 or Eic1 are absent. Interestingly, fission yeast Cnp3^CENP-C/Mif2^ is not essential indicating that it is not required for the propagation of Cnp1^CENP-A^ chromatin. Eic1 appears to perform an equivalent role to Mis18BP1^KNL2^ but associates with the essential constitutive CCAN components Mal2^CENP-O/Mcm21^, Fta7^CENP-Q/Okp1^ and Cnl2^Nkp2^, rather than Cnp3^CENP-C/Mif2^. Thus, it is possible that the highly conserved CCAN components CENP-O^Mal2/Mcm21^ and CENP-Q^Fta7/Okp1^ also function in other organisms to recruit CENP-A assembly factors; they may even have an equivalent underlying function that is redundant with CENP-C^Cnp3/Mif2^ at vertebrate centromeres.

In conclusion, our analysis has identified Eic1 as a factor that connects Mis16^RbAp46/48/Hat2^ and Mis18, which are required for Cnp1^CENP-A^ incorporation, with constitutive kinetochore components within the CCAN/Mis6/Ctf19 complex. We propose that Eic1 serves two major functions: (i) priming Cnp1^CENP-A^ assembly in concert with Mis16^RbAp46/48/Hat2^ and Mis18 and (ii) promoting kinetochore integrity in conjunction with the CCAN/Mis6/Ctf19 complex. The interdependency relationships among Eic1, Mis16^RbAp46/48/Hat2^ and Mis18, along with their similar dynamic localization to centromeres, indicate that Eic1, Mis16^RbAp46/48/Hat2^ and Mis18 form a complex that is temporarily released from centromeres during mitosis and can associate with centromeres independently of Cnp1^CENP-A^. The novel physical interaction via Eic1 that we have uncovered between constitutively bound CCAN components and Mis18 is likely to be fundamental for the temporal regulation of Eic1–Mis16^RbAp46/48/Hat2^–Mis18 association with centromeres, and thereby the cell-cycle-dependent assembly of Cnp1^CENP-A^ chromatin. The identification of Eic1 and Eic2 adds to the repertoire of known cell-cycle-regulated centromeric proteins involved in maintaining CENP-A at centromeres and provides a good example of how distinctly different proteins can contribute to a conserved cellular process in diverse organisms.

## 5. Material and methods

### 5.1. Yeast strains and standard techniques

Standard methods were used for fission yeast growth, genetics and manipulation [44]. Five-fold serial dilutions of the indicated strains were spotted onto YES media containing Phloxine B for growth assays, or DMSO or 12.5 μg ml^−1^ TBZ for TBZ sensitivity assays. Gene deletions, tagging and centromeric plasmid transformations were carried out by either the lithium acetate transformation method or electroporation. The *eic1^+^:hyg^R^* allele was generated by integrating a hygromycin resistance marker within the 3′UTR of *eic1^+^* at its endogenous locus. The *eic1-1* and *eic1-2* alleles were generated likewise, but derived by error-prone PCR using the GeneMorph II random mutagenesis kit (Agilent Technologies). The epitope-tagged alleles of Eic1, Eic2, Hat1, Fta7 and Cnl2 were generated at their respective endogenous loci by integrating an in-frame GFP, 3HA or 13myc cassette at their respective C termini [45].

### 5.2. Immunoaffinity purification and mass spectrometry

For Mis16 and Mis18 pulldowns, 5 g of pulverized *S. pombe* cells expressing Myc-tagged Mis16 or Mis18 were used for immunoprecipitation with anti-Myc antibody 9E10 (Covance) coupled to Protein G Dynabeads (Life Technologies), alongside an untagged control. For Eic1 and Eic2 pulldowns, 5 g of pulverized *S. pombe* cells expressing GFP-tagged Eic1 or Eic2 were used for immunoprecipitation with anti-GFP antibody A11122 (Life Technologies) coupled to Protein G Dynabeads, alongside an untagged control. After washes, Dynabeads with immunoprecipitated material were subjected to on-bead tryptic digestion, following which the samples were treated as described [9]. The average number of unique peptides corresponding to proteins that were reproducibly enriched in the epitope-tagged samples but consistently absent in the untagged controls, over three independent biological replicates, is presented.

### 5.3. Multiple sequence alignment

Orthologues of Eic1 and Eic2 among the *Schizosaccharomyces* species were identified by BLAST, PSI-BLAST or synteny searches against the *Schizosaccharomyces* group database available at the Broad Institute *Schizosaccharomyces* Comparative Genome Project [46]. Primary sequences of Eic1 or Eic2 orthologues were aligned using ClustalW and T-Coffee.

### 5.4. Co-immunoprecipitations and western analyses

For co-immunoprecipitation experiments, 2 g of pulverized *S. pombe* cells expressing the indicated epitope-tagged proteins were used for immunoprecipitation using anti-GFP antibody A11122 (Life Technologies), anti-Myc antibody 9E10 (Covance), anti-Myc antibody 9B11 (Cell Signaling) or anti-HA antibody 12CA5 (Roche) coupled to Protein G Dynabeads (Life Technologies). The same antibodies were also used for western analyses as indicated. Anti-Bip1 was used as a loading control where indicated.

### 5.5. Recombinant protein co-expression and binding assays

Codon-optimized ORFs of Eic1, Mis16 and Mis18 were synthesized by GeneArt (Life Technologies), for expression in *E. coli*. These were then sub-cloned into pGEX-6P-1 (GST) (GE Healthcare, gift from J. Welburn) or pEC(K)-3CHis (His) (gift from A. A. Jeyaprakash) expression vectors as indicated, and co-transformed into the BL21-Gold-pLysS *E. coli* strain (Agilent Technologies). Co-transformants were grown in SuperBroth supplemented with carbenicillin and kanamycin as appropriate, and the indicated proteins co-expressed by induction with 0.3 mM IPTG. GST pulldowns were done using glutathione agarose (Sigma). His pulldowns were done using Ni-NTA agarose beads (Qiagen). Samples were resolved on NuPAGE Bis-Tris gels (Life Technologies) and stained using InstantBlue (Expedeon).

### 5.6. Quantitative chromatin immunoprecipitation

The indicated *S. pombe* strains were grown in YES media at 32°C. If appropriate, cells were shifted to restrictive temperature (36°C) for 8 h, or continued to grow for the same length of time at permissive temperature (25°C) before fixation. For ChIPs on centromeric plasmids, cells harbouring pH-cc2 [38,47] were grown in PMG media minus adenine, minus uracil, at 32°C. To confirm that plasmids were behaving episomally and had not integrated, a plasmid stability test was performed at the time of fixation. Cells (100–1000) were plated onto YES supplemented with 1/10th adenine and allowed to form colonies. Samples exhibiting no integrations were used for ChIP.

ChIP was performed as described [9], or with the following modifications. Cells were fixed with 1% formaldehyde (Sigma) for 20 min at room temperature and lysed using a bead beater (Biospec Products). Cell lysates were sonicated in a Bioruptor (Diagenode) (15 min, 30 s On and 30 s Off at ‘High’ (200 W) position). For Cnp1^CENP-A^ ChIPs, anti-Cnp1^CENP-A^ antiserum was used with Protein G agarose beads (Roche). For all other ChIPs, Protein G Dynabeads (Life Technologies) were used along with anti-GFP A11122 (Life Technologies), anti-HA 12CA5 (Roche) or anti-Myc 9B11 (Cell Signaling), as appropriate. Immunoprecipitated DNA was recovered using the Chelex-100 resin (BioRad) [48]. ChIPs were analysed by real-time PCR using Lightcycler 480 SYBR Green (Roche) with primers specific to the central cores of centromere 2 (*cc2*) or centromeres 1/3 (*cc1/3*), the innermost repeats of centromere 1 (*imr*) or *act1*. All ChIP enrichments were calculated as % DNA immunoprecipitated at the locus of interest relative to the corresponding input samples, and normalized to % DNA immunoprecipitated at the *act1* locus. Histograms represent data averaged over at least three biological replicates. Error bars represent standard deviations from at least three biological replicates.

### 5.7. ChIP-seq analysis

*Schizosaccharomyces pombe* strains expressing the indicated GFP-tagged proteins were grown in YES media at 32°C to 1.25 × 10^9^ cells at a density of 1 × 10^7^ cells ml^−1^. Cells were fixed for 15 min in 1% formaldehyde (Sigma) and lysed with 0.4 mg ml^−1^ Zymolyase 100 T (AMS Biotechnology Europe) in PEMS for 1 h at 36°C. Cell lysates were sonicated in a Bioruptor (Diagenode) (20 min, 30 s On and 30 s Off at ‘High’ (200 W) position) and immunoprecipitated overnight using anti-GFP antibody A11122 (Life Technologies) and Protein G Dynabeads (Life Technologies). The samples were washed and cross-links reversed using 1% SDS for 4 h at 65°C. Immunoprecipitated DNA was recovered using a Qiagen PCR purification kit, and centromeric enrichment was verified by qPCR using Lightcycler 480 SYBR Green (Roche). Illumina libraries were prepared following the TruSeq Nano DNA kit (Illumina) guidelines using NEXTflex (Bio Scientific) adapters with internal barcodes. Multiplexed libraries were 100 bp paired-end sequenced on an Illumina HiSeq2000 (Ark Genomics, Edinburgh, UK). ChIP-seq data were mapped onto the *S. pombe* genome assembly EF2 (Ensemble) using Bowtie2. Coverage calculations and peak calling were done using MACS and PeakSplitter.

### 5.8. Cytology

Immunolocalization and microscopy were performed as described [9]. If appropriate, cells were shifted to restrictive temperature (36°C) for 8 h before fixation. Cells were fixed for 7–10 min with 3.7% formaldehyde (Sigma). Fixation of cells for tubulin staining used formaldehyde and 0.06% glutaraldehyde. Antibodies used were anti-GFP A11122 (1 : 200) (Life Technologies), TAT1 anti-tubulin (1 : 15) (gift from K. Gull) or anti-Cnp1^CENP-A^ antiserum (1 : 1000). Alexa Fluor 594- and 488-coupled secondary antibodies were used at 1 : 1000 (Life Technologies).

### 5.9. Centromeric plasmid selection system and stability

pH-cc2 (H denotes an *otr* heterochromatic element and cc denotes central domain DNA) carries *ura4^+^* and *sup3-5* (suppressor of *ade6-704*) selection systems [38,47]. Cells without *ura4^+^* cannot grow on minus-uracil plates, while *ade6-704* cells do not grow without adenine and form red colonies on 1/10th adenine plates. The *sup3-5*-tRNA gene suppresses a premature stop in *ade6-704*, allowing growth on minus-adenine plates. Cells containing pH-cc2 form a high percentage of white or sectored colonies on 1/10th adenine indicator plates, demonstrating their relative mitotic stability in wild-type cells. In cells lacking Clr4, however, their mitotic stability is lost due to a lack of heterochromatin-dependent centromeric cohesion. To confirm that plasmids were behaving episomally and had not integrated, a plasmid stability test was performed at the time of fixation for ChIP. Cells (100–1000) were plated onto YES supplemented with 1/10th adenine and allowed to form colonies. Samples exhibiting no integrations were used for ChIP.

## Note added in proof

A concurrent study has identified Eic1 and Eic2 as Mis19 and Mis20, respectively, in association with Mis18 [49].

## Supplementary Material

Subramanian-et-al_SuppInfo

## Data accessibility

ChIP-seq data have been submitted to the Gene Expression Omnibus under accession no. GSE 54685.
