# Supplementary material for: Eic1 links Mis18 with the CCAN/Mis6/Ctf19 complex to promote CENP-A assembly
Source: Open Biol. 2014 Apr 30;4(4):140043. doi: 10.1098/rsob.140043 (PMC4043117; doi:10.1098/rsob.140043)
Supplement: Subramanian-et-al_SuppInfo [file rsob140043supp1.pdf]

Eic1 links Mis18 with the CCAN/Mis6/Ctf19 complex to promote CENP-A assembly

Lakxmi Subramanian[1], Nicholas R.T. Toda[1], Juri Rappsilber[1] and Robin C. Allshire[1],*

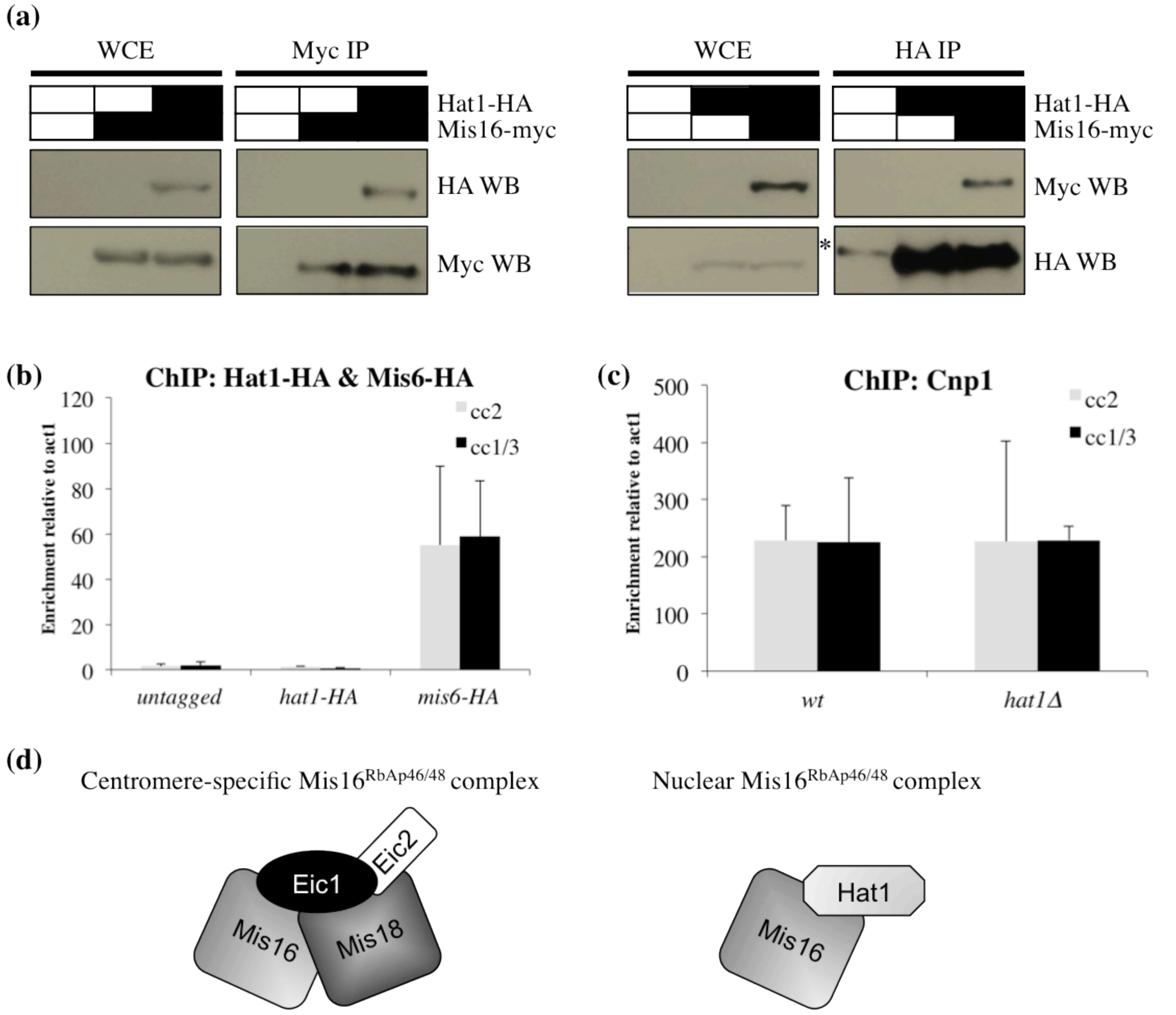

Figure S1. Hat1 does not affect Cnp1$^{CENP\text{-}A}$ association with centromeres. (**a**) Hat1-HA and Mis16-myc co-immunoprecipitate in reciprocal pulldowns. The asterisk (*) in the right hand side panel denotes the IgG heavy chain. (**b**) Hat1-HA is undetectable at the central domain of centromeres. qChIP analyses showing enrichments of Hat1-HA and Mis6-HA at cc2 or cc1/3, relative to the *act1* locus. (**c**) *hat1*Δ cells display no loss of Cnp1$^{CENP\text{-}A}$ at centromeres. qChIP analyses of Cnp1$^{CENP\text{-}A}$ association with centromeres in the indicated strains. Enrichment of cc2 or cc1/3 DNA relative to the *act1* locus is presented. Error bars in panels (**b**) and (**c**) represent standard deviation between at least three biological replicates. (**d**) Mis16$^{RbAp46/48/Hat2}$ likely participates in two distinct complexes: a centromere-specific complex in association with Mis18, Eic1 and Eic2 (left) and a more general nuclear complex in association with Hat1 (right).

(a)

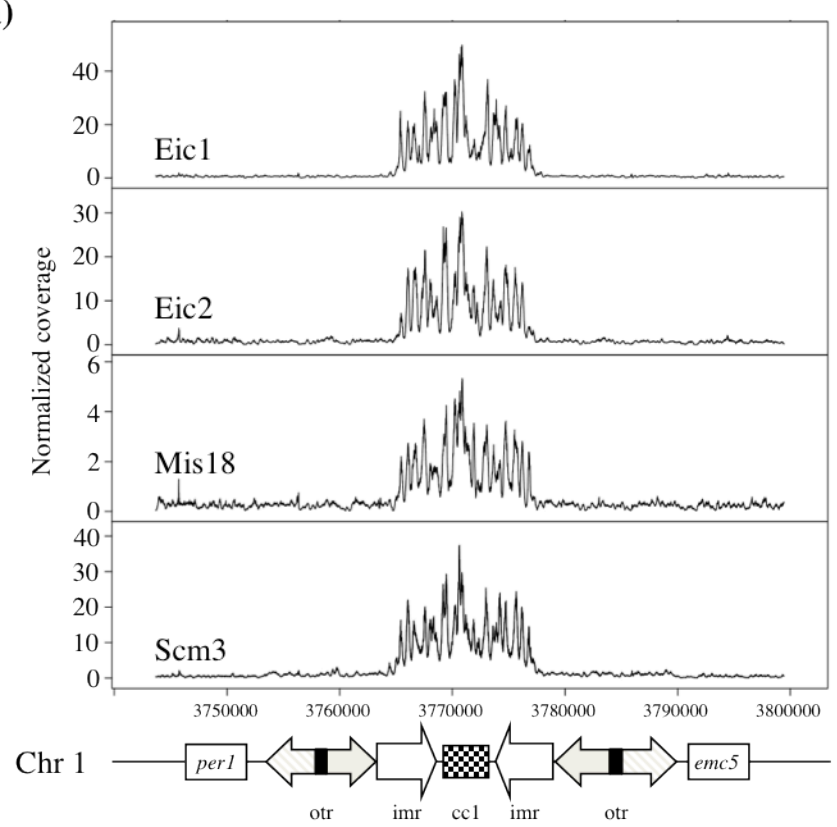
Normalized coverage
Eic1
Eic2
Mis18
Scm3
3750000
3760000
3770000
3780000
3790000
3800000
Chr 1
per1
emc5
otr
imr
cc1
imr
otr

(b)

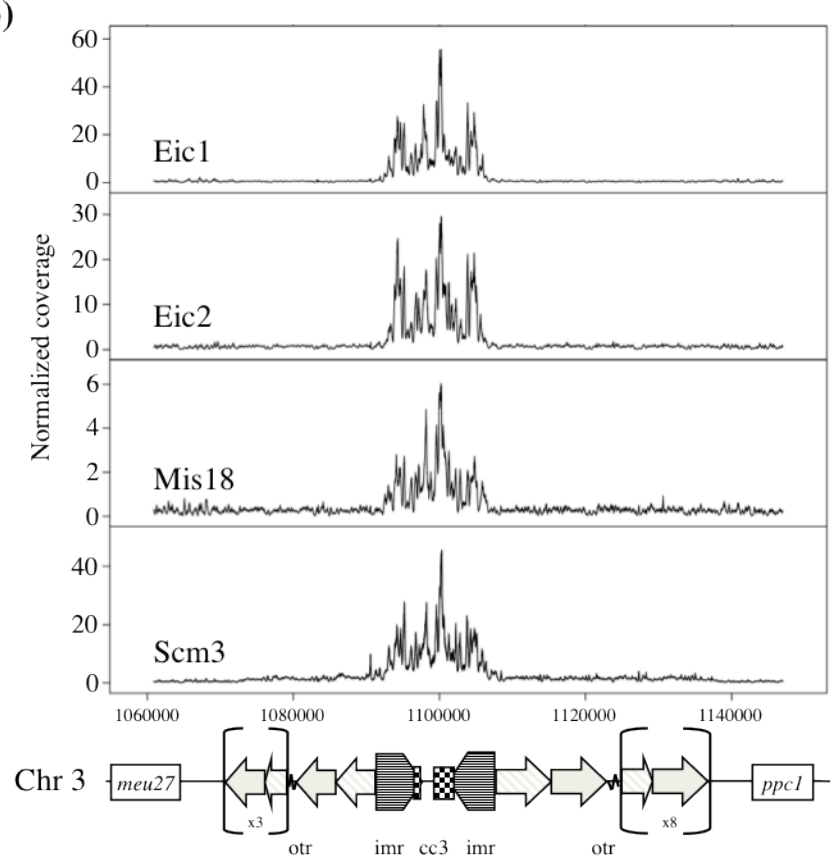
Normalized coverage
Eic1
Eic2
Mis18
Scm3
1060000
1080000
1100000
1120000
1140000
Chr 3
meu27
ppc1
x3
x8
otr
imr
cc3
imr
otr

Figure S2. Eic1 and Eic2 associate specifically with centromeres. (**a**) & (**b**) Eic1 and Eic2 exhibit very similar genome-wide association profiles as Mis18 and Scm3^{HJURP}, and associate specifically with the central domains of centromeres 1 & 3. The ChIP-seq profiles of GFP-tagged Eic1, Eic2, Mis18 and Scm3 across centromere 1 (**a**) and centromere 3 (**b**) are presented, alongside schematic diagrams of the respective centromeres (bottom). Normalized coverage represents the number of sequencing fragments obtained from anti-GFP IP normalized to that obtained from the input.

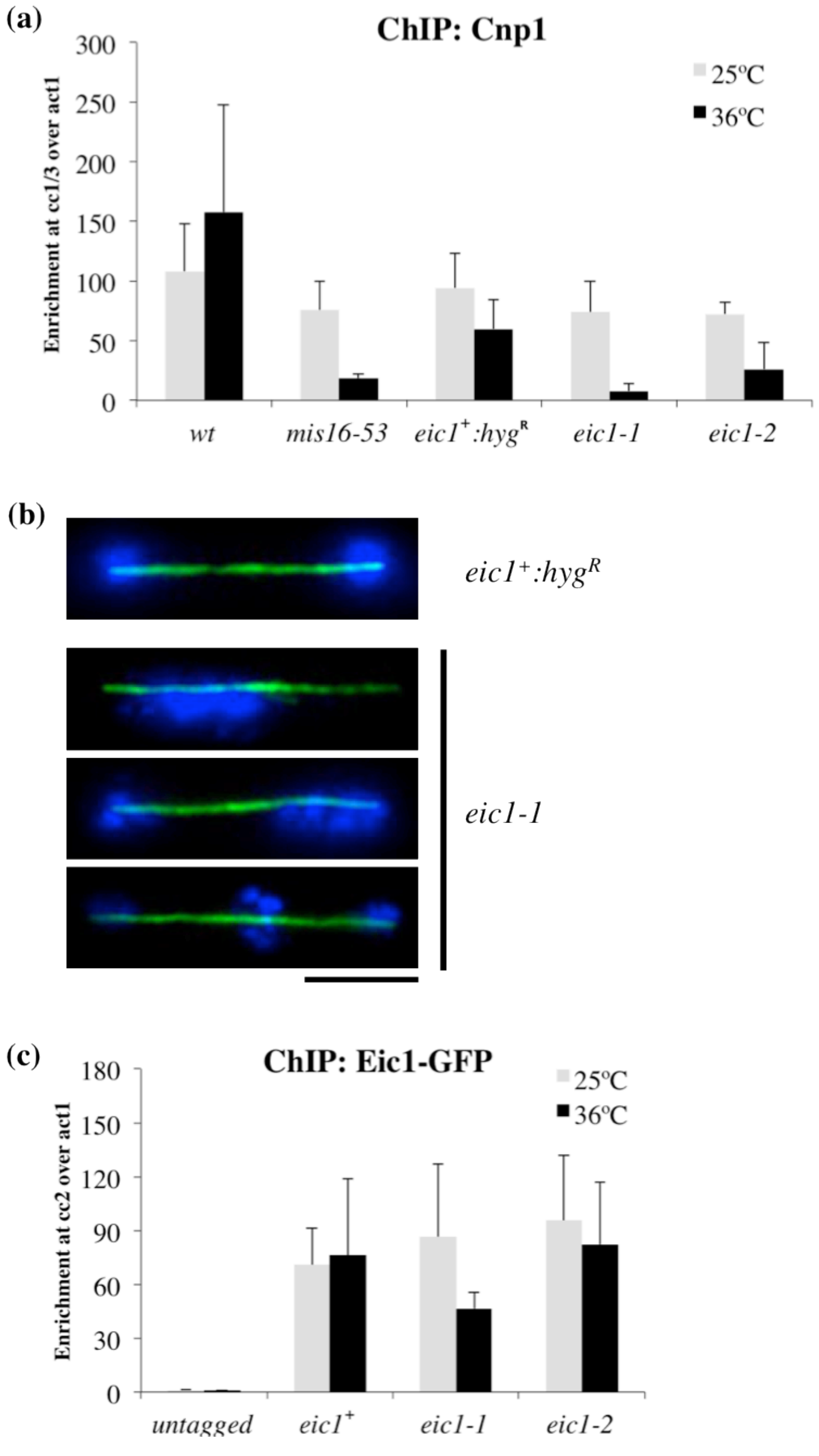

Figure S3. Disruption of Eic1 function affects Cnp1$^{CENP\text{-}A}$ chromatin assembly, and causes severe defects in chromosome segregation. (a) *eic1* mutants display reduced Cnp1$^{CENP\text{-}A}$ levels at centromeres. qChIP analyses of Cnp1$^{CENP\text{-}A}$ association with centromeres in the indicated strains when grown at permissive (25°C) vs restrictive temperature (36°C) for 8 hr. Enrichment of cc1/3 DNA relative to the *act1* locus is presented. (**b**) The *eic1-1* mutant exhibits severe defects in

chromosome segregation. Immunofluorescence of *eic1*$^{+}$*:hyg*^{R} or *eic1-1* cells shifted to restrictive temperature (36°C) for 8 hr, fixed and stained with antibodies to tubulin (TAT1) (green), and DAPI (blue). Cells shown are in anaphase. Representative images showing hypercondensed DNA, unequal DNA segregation or lagging chromosomes in *eic1-1* are presented. Scale bar, 5μm. (**c**) GFP tagged Eic1-1 and Eic1-2 mutant proteins associate with centromeres even at restrictive temperature (36°C). qChIP analyses of Eic1-GFP (wild-type or mutant) association with centromeres in the indicated strains when grown at permissive (25°C) vs restrictive temperature (36°C) for 8 hr. Enrichment of cc2 DNA relative to the *act1* locus is presented. Error bars in panels (**a**) and (**c**) represent standard deviation between at least three biological replicates.

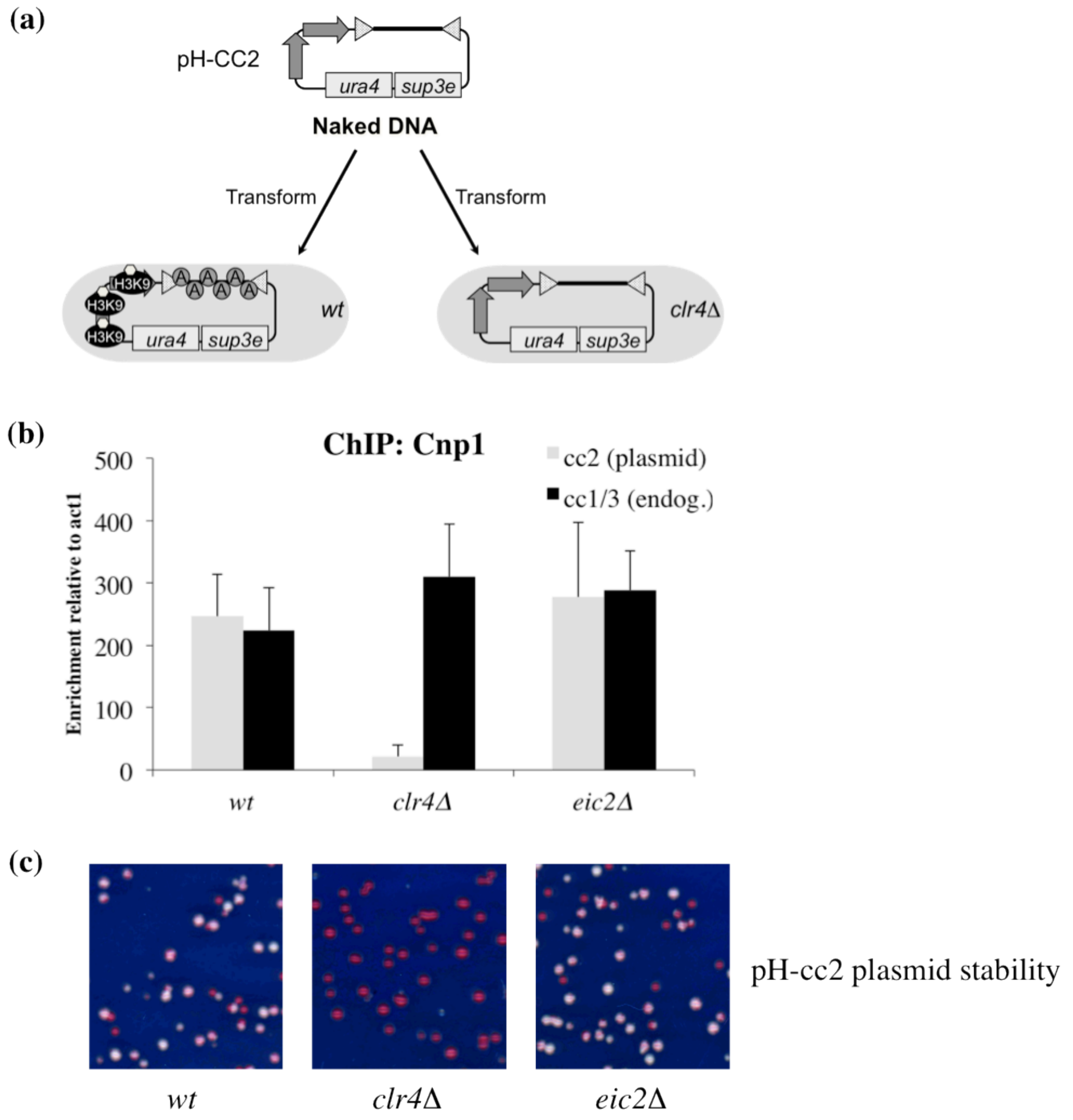

Figure S4. Eic2 is dispensable for Cnp1^{CENP-A} establishment on naïve centromeric DNA. (a) A schematic representation of the Cnp1^{CENP-A} establishment assay on the centromeric pH-cc2 plasmid [38]. (**b**) Cnp1^{CENP-A} establishment is unaffected in *eic2*Δ cells. qChIP analyses of Cnp1^{CENP-A} association with the centromeric plasmid pH-cc2 and endogenous centromeres, in the indicated strains transformed with pH-cc2. Enrichment of cc2 (plasmid) or cc1/3 (endogenous) DNA relative to the *act1* locus is presented. Error bars represent standard deviation between at least three biological replicates. (**c**) Centromeric plasmid stability is retained even in the absence of Eic2. An aliquot of cells used in (**b**) were plated on YES media supplemented with 1/10th adenine to assess plasmid loss. *eic2*Δ cells do not show any obvious defects in plasmid stability, they form sectored colonies at a high frequency much like wild-type.

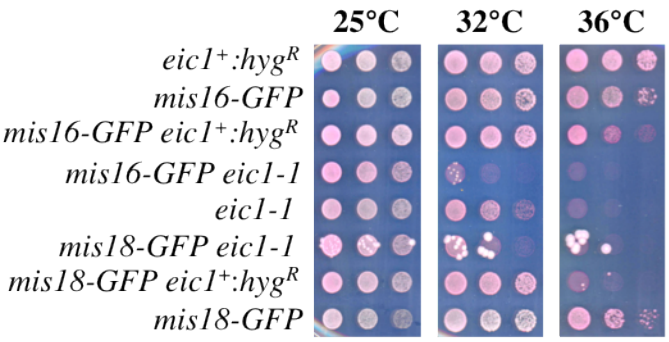

Figure S5. *mis16-GFP* & *mis18-GFP* exhibit synthetic genetic interactions with *eic1*. Five-fold serial dilutions of the indicated cells spotted on YES+Phloxine B media and incubated at the indicated temperatures; dead cells stain dark pink.

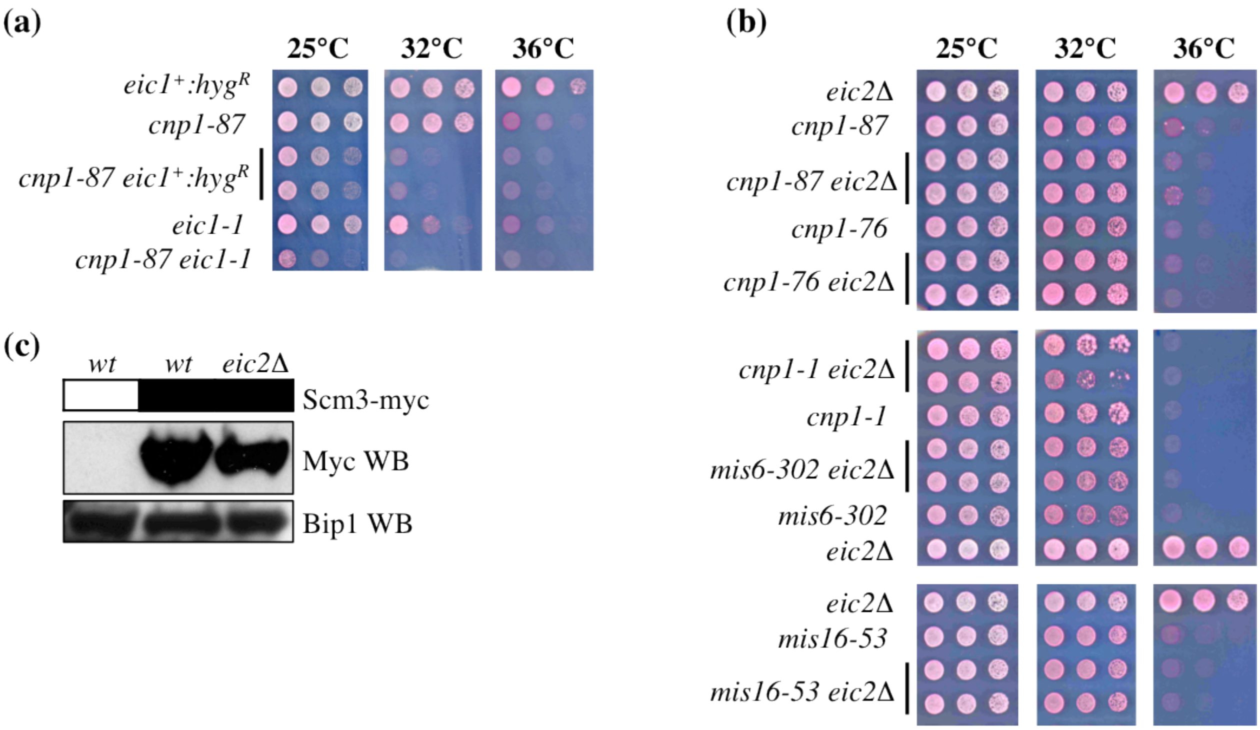

Figure S6. Analysis of genetic interactions between *eic1* or *eic2* mutants and mutations in Cnp1$^{CENP\text{-}A}$ or Cnp1$^{CENP\text{-}A}$ assembly factors. (**a**) *eic1-1* cells display reduced growth when combined with *cnp1-87*. (**b**) *eic2*Δ cells display no obvious growth defects when combined with mutations in *cnp1, mis6* or *mis16*. Five-fold serial dilutions of cells spotted on YES+Phloxine B media and incubated at the indicated temperatures; dead cells stain dark pink. (**c**) Scm3-myc protein levels remain unaffected in *eic2*Δ cells. Whole cell extracts of the indicated strains were resolved on an SDS-PAGE gel and subjected to Western analysis using either an anti-Myc (top) or an anti-Bip1 antibody (bottom).

(a)

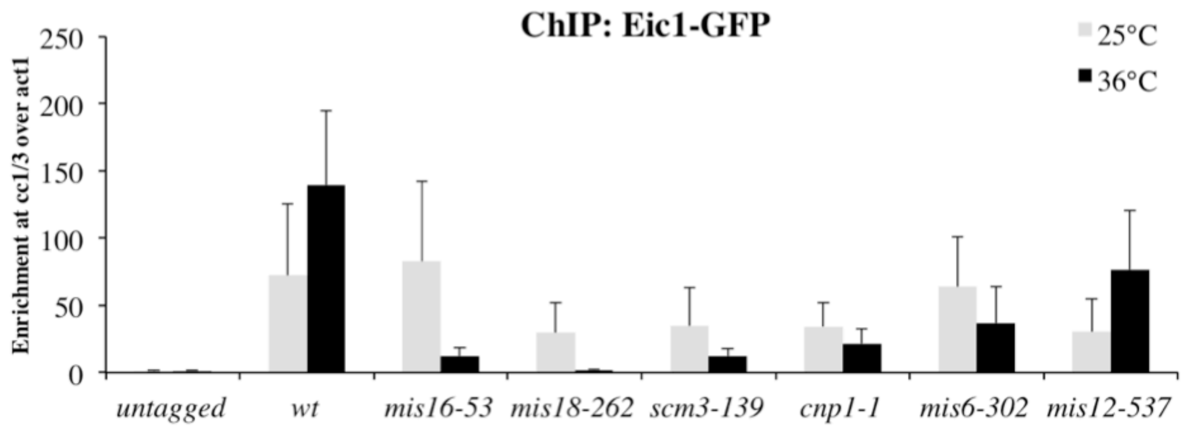

(b)

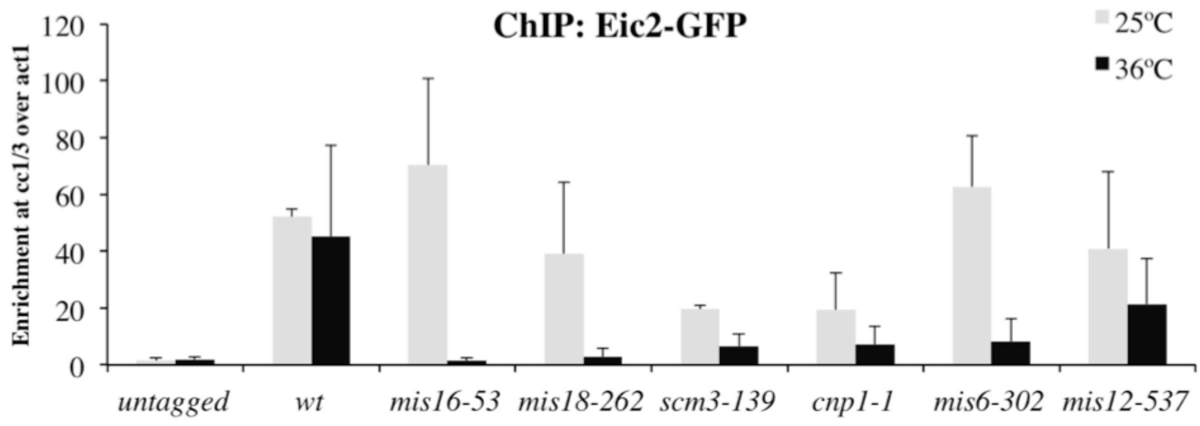

Figure S7. Eic1 and Eic2 depend on distinct Cnp1$^{CENP\text{-}A}$ assembly factors for their association with centromeres. (**a**) Eic1 association with centromeres is dependent on Mis18, Mis16$^{RbAp46/48/Hat2}$ and Scm3^{HJURP}, but is largely independent of Cnp1$^{CENP\text{-}A}$, Mis6$^{CENP\text{-}I/Ctf3}$ and Mis12. qChIP analyses of Eic1-GFP association with centromeres in the indicated strains when grown at permissive (25°C) vs restrictive temperature (36°C) for 8 hr. (**b**) Eic2 association with centromeres is dependent on Mis18, Mis16$^{RbAp46/48/Hat2}$, Scm3^{HJURP}, Cnp1$^{CENP\text{-}A}$, Mis6$^{CENP\text{-}I/Ctf3}$ and Mis12. qChIP analyses of Eic2-GFP association with centromeres in the indicated strains when grown at permissive (25°C) vs restrictive temperature (36°C) for 8 hr. Enrichment of cc1/3 DNA relative to the *act1* locus is presented. Error bars represent standard deviation between at least three biological replicates.
